# THE IMPACT OF THE COVID-19 PANDEMIC ON INFORMAL CAREGIVERS OF PERSONS WITH DEMENTIA IN THE POST-VACCINATION ERA

**DOI:** 10.1093/geroni/igad104.1771

**Published:** 2023-12-21

**Authors:** Sarah Bannon

**Affiliations:** Mount Sinai Hospital, New York City, New York, United States

## Abstract

The COVID-19 pandemic disproportionately impacted individuals living with dementia and their family care-partners. Prior to the pandemic, many family care-partners had insufficient support to manage stressors and experienced resultant negative physical and mental consequences. Systematic reviews of studies from the early pandemic suggest that circumstances dramatically worsened for family care-partners’, though it is unclear whether and how they were impacted by ongoing fluctuations in COVID-19 risk for infection and safety guidelines following the availability of vaccines. Using qualitative design informed by a stress-and-coping theoretical framework, our objective was to characterize the ongoing impact of the pandemic on family care-partners of persons living with dementia. We used purposive sampling to recruit participants from a larger survey study of dementia caregiving during the COVID-19 pandemic. Study participants (n=18) completed a 1-hour virtual interview with that was transcribed, de-identified, and analyzed using thematic analysis. We identified 4 overarching themes characterizing family caregivers’ stressors that included: 1) changes in care, routines, and support; 2) actual or feared COVID-19 infection; 3) changes in the functioning and health of care-partners and care-recipients; 4) experiences with care facilities care transitions. We also identified 4 overarching themes characterizing care-partners’ coping strategies, including: 1) care-partner resourcefulness; 2) participation in meaningful, enjoyable, and restorative activities; 3) personal outlook and attitudes to reduce distress; and 4) connection with specific formal and informal supports. Our findings underscore the continued challenges that family care-partners experience and highlight important skills, supports, and resources to mitigate negative health outcomes and other long-term consequences.

